# Profiles of Perfectionism Among Adolescents Attending Specialized Elite- and Ordinary Lower Secondary Schools: A Norwegian Cross-Sectional Comparative Study

**DOI:** 10.3389/fpsyg.2019.02039

**Published:** 2019-09-06

**Authors:** Annett Victoria Stornæs, Jan H. Rosenvinge, Jorunn Sundgot-Borgen, Gunn Pettersen, Oddgeir Friborg

**Affiliations:** ^1^Department of Sports Medicine, Norwegian School of Sport Sciences, Oslo, Norway; ^2^Department of Psychology, University of Tromsø, The Arctic University of Norway, Tromsø, Norway; ^3^Department of Health and Care Sciences, University of Tromsø, The Arctic University of Norway, Tromsø, Norway

**Keywords:** perfectionism, adolescents, latent class analysis, subgroups, mental health

## Abstract

The versatile construct of perfectionism has been heavily debated, e.g., its nature or measurement constituents, how it influences performances or, most importantly, our health. Conventional linear analyses seem inadequate to address such challenges. Hence, we used a latent variable and a person-centered approach to identify different patterns of perfectionism, and their relationships with psychological health as outcome among early adolescents (13–14 years) attending conventional or elite sports-/performance-oriented lower secondary schools (14 schools, 832 students, 53% girls). All students completed two perfectionism scales, i.e., the child-adolescent perfectionism scale (CAPS) and the frost multidimensional perfectionism scale (FMPS). The criterion-related variables of psychological health included anxiety, depression, eating disorder problems, self-worth and resilience, respectively. Exploratory and confirmatory factor analyses yielded a four-factor representation of perfectionism. Using latent class analysis extracted five profiles of perfectionism, which were related to the criterion variables. Three profiles were clear indicators of either low or high perfectionism score patterns. Two profiles showed a mixed picture of high and low scores, whereas one represented a psychological healthy subgroup. About four of ten adolescents in the ordinary schools matched the two most debilitating perfectionism profiles compared to two of ten in the elite schools. How these results align with international findings is discussed along with the relevance for early interventions aimed at preventing the potential downsides of perfectionism. Longitudinal studies are neeed to explore profile trajectories as well as possible health consequences.

## Introduction

According to a recent meta-analysis (Curran and Hill, 2017) youth’s perfectionism levels have steadily increased the last 25–30 years. Perfectionism is a multidimensional, intra- and interpersonal construct consisting of exceedingly high or unrealistic personal standards, accompanied by overly self-critical evaluations (Frost et al., 1990; Hewitt and Flett, 1991). Two interrelated superordinate dimensions have been identified, i.e., “perfectionistic concerns” (or “evaluative concerns perfectionism”) and “perfectionistic strivings” (or “personal standard perfectionism”) (Frost et al., 1993; Dunkley et al., 2000; Stoeber and Otto, 2006). People with perfectionistic concerns tend to be preoccupied with a fear of making mistakes, a fear of negative evaluations from others and that significant others are holding rigorous standards for them (Dunkley et al., 2000; Gotwals et al., 2012). Hardly surprising then, such concerns run along with poor mental health among adults (Hill and Curran, 2016; Limburg et al., 2017), and among adolescents in terms of outcomes like anxiety, depression and eating disorder symptoms (Hewitt et al., 2002; Bento et al., 2010; Flett et al., 2011). A similar consistency is, however, not evident between mental health and “perfectionistic strivings”. Thus both adaptive and maladaptive outcomes have been linked to the personal standards and self-oriented strivings toward perfection (Stoeber and Otto, 2006; Gotwals et al., 2012; Jowett et al., 2016; Hill et al., 2018). Different health outcomes raise the issue of how dimensions or facets of perfectionism are related. This issue is further relevant considering the fact that distinct profiles emerge when subdimensions of perfectionism are analyzed together, and such profiles are differently related to health indicators (Boone et al., 2010; Hill, 2013; Sironic and Reeve, 2015; Gustafsson et al., 2016). In addition, health outcomes may be moderated by contextual aspects like for instance students’ type of school settings. Hence, certain facets of perfectionism may be more prominent and endanger health to a greater extent within elite or high performance and sport contexts than in low performance contexts.

At least three analytical approaches have been used to identify perfectionism profiles. The first one is the *a priori* 2 × 2 model of perfectionism isolating four within-person subgroups based on the higher order factors “perfectionistic strivings” and “perfectionistic concerns” (Gaudreau and Thompson, 2010; Gaudreau, 2012, 2016; Hill, 2013). Secondly, profiles of perfectionism have been derived from cluster analyses, whereas the profiles may differ in terms of maladaptive outcomes depending on gender and performance contexts (Dixon et al., 2004; Vallance et al., 2006; Boone et al., 2010). A third approach is latent class analysis (LCA). Similar patterns of differences between perfectionism subgroups and mental health have emerged irrespective of these three analytical approaches (Dixon et al., 2004; Boone et al., 2010; Gotwals, 2011; Cumming and Duda, 2012; Hill, 2013; Damian et al., 2014; Sironic and Reeve, 2015). These approaches are, however, not equally adequate. In contrast to a cluster analysis, the LCA approach is a more complex, robust and stable approach which is model based, and with more stringent criteria to determine the final profile model (Pastor et al., 2007; Marsh et al., 2009). Moreover, a LCA approach may give more nuanced knowledge of how various perfectionism profiles in adolescents are linked to adaptive and maladaptive indicators of mental health, beyond traditional variable-centered approaches where mental health outcomes are linked to each separate perfectionism dimension (Pastor et al., 2007).

Among adolescents one LCA study identified six distinct classes of perfectionism (Sironic and Reeve, 2015). A “mixed maladaptive perfectionism” profile included high ratings on all dimensions. Male and female high-school students with this profile reported higher levels of anxiety, depression and stress compared to the other perfectionism subgroups. The remaining profiles comprised an “externally motivated maladaptive” subgroup with low personal standards and high scores for perfectionism prescribed by significant others, and concerns and doubts about their own performances. An “adaptive” profile with high personal standards and low externally factor scores has also been identified, along with two non-perfectionism groups and one subgroup of students that exclusively valued order and organization (Sironic and Reeve, 2015). Furthermore, based on perfectionism scores and parental climate scores four latent profiles have been identified in adolescent athletes (Gustafsson et al., 2016).

A large number of perfectionism studies in sports have comprised mainly athlete boys (Vallance et al., 2006; Hill et al., 2008; Stoeber et al., 2009; Appleton and Hill, 2012; Hill, 2013; Madigan et al., 2016, 2017; Hill et al., 2018). Moreover, perfectionism profiles have been studied among adolescent athletes (Hill, 2013) and ordinary school students (Boone et al., 2010; Sironic and Reeve, 2015) separately. Thus, there is a lack of comparative studies, and a gap of knowledge about perfectionism profiles across gender and within a broder range of high-performance contexts, i.e., boys and girls attending ordinary versus specialized school contexts for talented athletes or performing artists. The relevance of filling this gap of knowledge rests on the importance of identifying profiles of perfectionism that may constitute a risk of poor health among young adolescents in a vulnerable developmental stage. Such risks may be particularly important to contrast with students in ordinary schools, because adolescents attending specialized elite schools may have to face environments and contexts where high goals of achievements and performances are highly valued, yet hard to cope with (Hall and Hill, 2012; Bergeron et al., 2015; Hewitt et al., 2017). To expand on the previous research the present study aims to:

(1) Examine the factor structure across the items of two commonly used measures of perfectionism.
(2) Identify meaningful profiles of perfectionism generated from the perfectionism factor scores.
(3) Examine possible differences in the proportion of girls and boys from specialized- and ordinary schools within each of the profiles of perfectionism.
(4) Examine the differences of the identified perfectionism profiles in terms of mental health and psychological functioning.

## Materials and Methods

### Participants

The participants in this cross-sectional survey consisted of Norwegian students aged 13–14 years who were enrolled into 8th grade at 14 lower secondary schools during the school year 2015/2016. Students (*n* = 1055) from 11 ordinary schools were eligible. To ensure sociodemographic representativity the ordinary schools were randomly drawn from regions within two of the largest counties in the Eastern part of Norway. Also eligible were students (*n* = 199) at all the three national private elite lower secondary sport schools, and the two elite classes for performing arts (ballet and music) located at the ordinary public schools in the catchment area. From the total sample (*n* = 1254) students were excluded due to missing or inadequate parental consent (*n* = 95) or survey completion (*n* = 19). In addition 308 students did not participate for unknown reasons, thus yielding a final sample of 832 students. Of these, 166 students (82 girls and 84 boys) came from the elite schools and classes, and 666 students from ordinary schools (361 girls and 305 boys). The response rate for the two samples was 83 and 63%, respectively.

### Procedure

The consenting schools appointed a teacher or staff member as the contact person to the research group. Study information were distributed to the students and their guardians separately, and both guardians and students had to provide their written informed consent. Additionally, the first author informed all students at school about the study purpose ahead of and at the day of data collection. Students completed questionnaires during one school hour with the presence of a research group member.

### Self-Report Measures

In the present study the internal consistency (Cronbach’s α) ranged from 0.67–0.95 (Table 1).

**TABLE 1:** Descriptive Statistics and Correlations Between the Measured Study Variables.

**Variable**	***M***	**(SD)**	**1**	**2**	**3**	**4**	**5**	**6**	**7**	**8**	**9**	**10**	**11**	**12**	**13**
1 FMPS PS	2.97	(0.89)	α = 0.84												
2 FMPS CM	2.31	(0.77)	0.58	α = 0.82											
3 FMPS DA	2.78	(0.88)	0.39	0.54	α = 0.67										
4 FMPS PE	2.22	(0.93)	0.34	0.47	0.31	α = 0.82									
5 FMPS PC	1.76	(0.79)	0.10	0.43	0.32	0.60	α = 0.68								
6 FMPS O	3.88	(0.76)	0.46	0.20	0.17	0.05	–0.13	α = 0.8 4							
7 CAPS SOP	3.15	(0.74)	0.72	0.64	0.42	0.34	0.15	0.35	α = 0.86						
8 CAPS SPP	2.37	(0.82)	0.42	0.57	0.40	0.69	0.51	0.08	0.52	α = 0.87					
9 ANX	10.21	(6.78)	0.25	0.50	0.49	0.20	0.31	0.09	0.29	0.33	α = 0.86				
10 DEP	6.92	(5.03)	0.20	0.48	0.48	0.24	0.37	–0.06	0.24	0.38	0.74	α = 0.85			
11 WCSC	1.51	(1.57)	0.12	0.35	0.32	0.16	0.24	–0.01	0.19	0.27	0.55	0.56	α = 0.95		
12 READ	3.94	(0.55)	0.08	–0.25	–0.23	–0.19	–0.37	0.36	–0.03	–0.29	–0.35	–0.53	–0.37	α =0.92	
13 Glob. SW	3.19	(0.68)	–0.14	–0.43	–0.38	–0.17	–0.34	0.13	–0.20	–0.35	–0.58	–0.70	–0.67	0.62	α = 0.88

*Correlations above 0.08 were significant at *p* < 0.05, and below −0.125 and above 0.125 at *p* < 0.01. α, Cronbach’s alpha; CM, Concern over mistakes; DA, Doubts about actions; Glob. SW, Global Self-Worth from SPPA-R; O, Organization; PE, Parental Expectations; PC, Parental Criticism; PS, Personal Standards; SOP, Self-Oriented Perfectionism; SPP, Socially Prescribed Perfectionism; ANX, Anxiety; DEP, Depression; WCSC, EDE-Q Weight Concern and Shape Concern; READ, Resilience Scale for Adolescents.*

#### Frost Multidimensional Perfectionism Scale (FMPS)

The frost multidimensional perfectionism scale (FMPS) consists of 35 items covering six primary factors (Frost et al., 1990) that are typically combined in two over-arching dimensions: (a) “personal standards”; having exceedingly high standards for performances, and “organization”; emphasis on neatness, order and organization, and (b) “concern over mistakes”; worry about own performances, “doubt about actions”; a sense to doubt the quality of one’s performances, “parental expectations”; a strong integration of parents’ high expectations for performance, and “parental criticism”; worry of parental criticism, disapproval and loss of parental support. Items are rated on a five-point Likert scale ranging from 1 “strongly disagree” to 5 “strongly agree”. The subscale scores were calculated as the mean of all subscale items. In the present study we initiated both an explorative and a confirmatory factor analysis because previous psychometric studies (Stöber, 1998; Stumpf and Parker, 2000; Cox et al., 2002; Hawkins et al., 2006; Sironic and Reeve, 2015) have lend mixed support to the original factor model, and a loosely defined “organization” factor (Frost et al., 1990).

#### Child Adolescent Perfectionism Scale (CAPS)

The child adolescent perfectionism scale (CAPS) (Flett et al., 2000) is derived from the Hewitt and Flett Multidimensional Perfectionism Scale for adults (Hewitt and Flett, 1991), and measures the two dimensions “self-oriented perfectionism” (SOP, 12 items) and “socially prescribed perfectionism” (SPP, 10 items). SOP indicates excessively high personal standards and a need to fulfill them, whereas SPP imply the conviction that other people require perfection from oneself. The items are rated on a five-point Likert scale from false (1), neutral (3) to very true (5). Three items (SOP10, SPP20, and SOP22) were reversed to enable a mean subscale score from all items. In contrast to the FMPS, a Norwegian version of the CAPS did not exist. Thus, the CAPS was bi-directionally translated. The original (Flett et al., 2000) item numbers used in the present study diverge from later versions (Flett et al., 2016). Despite adequate support of the CAPS factor model (Sironic and Reeve, 2015; Flett et al., 2016; Leone and Wade, 2018), incongruent findings exist (McCreary et al., 2004; O’Connor et al., 2009).

#### Revised Children’s Anxiety and Depression Scale (Short Version) (RCADS-25)

The RCADS measures DSM-IV relevant anxiety and depressive symptoms in children (Chorpita et al., 2000). The short version, RCADS-25 (Ebesutani et al., 2012) encompasses two subscales; a general anxiety score (15 items) and a depression score (10 items). The items are rated on a four-point Likert scale from 0 “never” to 3 “always”. The subscale scores were calculated as the sum of all subscale items, and higher scores represent greater severity of anxiety and depression symptoms.

#### Eating Disorder Examination-Questionnaire (EDE-Q-11)

The EDE-Q-11 (Friborg et al., 2013) is derived from the 28-item EDE-Q (6.0) (Fairburn, 2009), and consists of 11 items measuring the importance of weight and shape concern (WCSC) for one’s self-worth. The items are rated on a seven-point scale from 0 “not at all” or “no days” to 6 “very much” or “all days”. The subscale scores were calculated as the mean of the subscale.

#### Resilience Scale for Adolescents (READ)

The READ (Hjemdal et al., 2006) consists of 28 items to assess the five protective factors “personal competence,” “social competence,” “structured style,” “family cohesion,” and “social resources.” All items were rated on a five-point Likert scale from 1 “strongly disagree” to 5 “strongly agree” (higher scores; more protection). As the READ subscales correlate strongly (Hjemdal et al., 2006), a mean score from all subscales was calculated.

#### Harter’s Self-Perception Profiles for Adolescents – Revised (SPPA-R)

One of the six subscales from the Norwegian short version of SPPA-R (Wichstrøm, 1995) was used. The subscale measures global self-worth as the evaluation of how much general value one places on oneself. The five items are rated on a four-point Likert scale; describes me: 1 “very poorly”, 2 “quite poorly”, 3 “quite well”, and 4 “very well”. Two negatively worded items were recoded to calculate a mean subscale score. Higher scores represent better global self-worth.

### Ethics Statement

The study was approved by the Regional Committee for Medical and Health Science Research Ethics (REC) in Southern Norway (project nr.2015/1358), and has been conducted in accordance to ethical guidelines, and the health research legislations and regulations.

### Statistical Analyses

The analyses were conducted in four steps: (1) principal component analysis (PCA) and confirmatory factor analyss (CFA) were used to explore an adequate perfectionism measurement model, (2) identification of subgroups of perfectionism using latent class analyses (LCA), (3) identification of the proportion of gender and school group within each of the perfectionism classes (profiles) using cross tabulation and (4) multivariate analyses of variance examining the differences of the identified perfectionism classes (or profiles) in terms of mental health and psychological functioning.

The PCA was performed on both perfectionism scales (FMPS and the CAPS), first separately and then combined, as they are distinct scales with mixed support for the number of factors (Stöber, 1998; Cox et al., 2002; Sironic and Reeve, 2015). The number of components were decided using the Kaiser’s criterion (eigenvalues > 1) and Horn’s parallel analysis, preferring the latter if deviant. The parallel analysis retains components with eigenvalues higher than the corresponding component eigenvalue from a randomly generated dataset. Components with ≤3 items were not retained. Items with small (<0.4) loadings, or with substantial (>0.5) cross-loadings, or with small differences between two or more component loadings (e.g., a primary loading of 0.55, and a cross loading of 0.4), were discarded. Loadings were Promax rotated (kappa = 4).

The sample was randomly split in two equal halves for the factor analysis, where the second half was used (CFA) to cross-validate the PCA findings. The CFA model fit were evaluated by the comparative fit index (CFI), the tucker-lewis index (TLI), chi-square difference test, the root mean square error of approximation, (RMSEA), and the standardized root mean square residual (SRMR). For the CFI and TLI, values > 0.95 should be preferred, but values about 0.90 are acceptable. RMSEA values < 0.05/0.06 are preferable (Hu and Bentler, 1999), while values between 0.05–0.08 indicate mediocre fit (MacCallum et al., 1996). SRMR < 0.08 are commonly considered a good fit (Hu and Bentler, 1999). Factor scores following the CFA modeling was saved and used in the following LCA.

The LCA was applied to identify subgroups of perfectionism based on the saved factor scores. A key challenge with fitting LCA models is to decide the number of classes (or subgroups) that is necessary to fit in order to adequately account for the correlations between the factor scores. We relied on the log likelihood ratio (LL), Akaike’s information criterion (AIC), Bayesian information criterion (BIC), and adjusted BIC (aBIC). Smaller values indicate a better fitting model preferring the BIC/aBIC as they require a more substantial improvement in fit than LL/AIC for retaining more complex models. We terminated adding subgroups when noticeably improvement in fit declined. The entropy index is additionally reported to measure the accuracy (0 = terrible, 1 = perfect classification) of the categorization of subjects into latent classes. The number of cases within each class was also of importance.

The final analyses used multivariate analysis of variance (MANOVA) to examine how well the retained classes, as representatives of the different perfectionism profiles, differed on a combined set of outcome variables (i.e., anxiety, depression, EDE-Q shape and weight concern, resilience and self-worth). Follow-up tests of a significant overall MANOVA effect were conducted with a univariate analysis for each outcome variable using the Scheffe’s test to adjust for the *post hoc* multiple comparisons.

All latent variable analyses were conducted using Mplus version 8.0 (Muthén and Muthén, 1998–2017), whereas the remaining principal component and multivariate analyses were conducted in SPSS Statistics version 24 (IBM SPSS Inc., Chicago, IL, United States).

## Results

### Descriptive Findings

The descriptive statistics, measurement reliabilities and correlations between the questionnaire variables are reported in Table 1. The CAPS and the FMPS subscales were strongly related, except with the FMPS organization subscale.

### Exploration and Confirmation of an Adequate Perfectionism Measurement Model

#### PCA of the FMPS

The PCA extracted six components with eigenvalues > 1 (8.57, 4.97, 1.94, 1.78, 1.22, 1.03; *R*^2^ = 0.56); however, we preferred the parallel analysis solution of four components as the fifth eigenvalue were lower than the random based eigenvalue of 1.41. This solution combined the two parental subscales “parental expectations” and “parental criticism” (named PEC), as well as the two subscales “concerns over mistakes” and the “doubts about actions” (named CMDA). Three items dropped out due to component misplacement or cross-loadings. This solution had acceptable loadings (0.42–0.95) and accounted for 51.1% of the variance (Supplementary Table X1).

#### PCA of the CAPS

The PCA extracted three components with eigenvalues > 1 (7.42, 2.49, and 1.39; *R*^2^ = 0.51); however, we retained two components as the third had three items, in which all originally had reverse wording (i.e., SOP10, SOP22 and SPP20). In the subsequent PCA two components were extracted, and two items dropped out due to a weak loading (i.e., SPP20) or cross-loading (i.e., SPP18). The final two-component solution showed acceptable item loadings (0.42–0.88) and accounted for 47.1% of the variance (Supplementary Table X2).

#### PCA Analysis of the FMPS and CAPS Combined

Kaiser’s criterion extracted 10 components (*R*^2^ = 0.61), but three components had too few items. The parallel analysis retained four components (*R*^2^ = 0.47). This solution had three items with component misplacement (i.e., SOP19, PS16, and SOP6); hence, these were removed from the final CFA analyses. Table 2 presents the final solution that combined the CAPS “socially prescribed perfectionism” and the FMPS “parental expectations/criticism” subscales, as well as the CAPS “self-oriented perfectionism” and the FMPS “personal standards” subscale. The components were labeled as (1) socially prescribed perfectionism/parental expectations and criticism (SPPEC), (2) self-oriented perfectionism/personal standards (SOPS), (3) concerns over mistakes and doubts about actions (CMDA), and (4), organization (O) (Table 2).

**TABLE 2:** Principal Component Analysis of the FMPS and the CAPS.

			**1**	**2**	**3**	**4**
	**Item**		**SPPEC**	**SOPS**	**CMDA**	**O**
CAPS	My family expects me to be perfect.	SPP8	**0.89**	0.05	–0.17	–0.02
FMPS	My parents set very high standards for me.	PE1	**0.83**	0.00	–0.12	0.15
FMPS	My parents wants me to be the best at everything.	PE11	**0.79**	0.17	–0.19	–0.04
CAPS	There are people in my life who expect me to be perfect.	SPP5	**0.76**	0.02	–0.02	0.05
FMPS	My parents have expected excellence from me.	PE20	**0.75**	–0.02	–0.10	0.16
FMPS	My parents have always had higher expectations for my future than I have.	PE26	**0.70**	–0.16	0.01	–0.13
CAPS	Other people always expect me to be perfect.	SPP13	**0.68**	0.11	0.01	–0.01
CAPS	People expect more from me than I am able to give.	SPP9	**0.58**	–0.09	0.24	0.00
CAPS	I feel that people ask too much of me.	SPP3	**0.58**	0.17	–0.05	0.12
FMPS	I never feel like I can meet my parents’ standards.	PC35	**0.57**	–0.22	0.23	–0.16
FMPS	Only outstanding performance is good enough in my family.	PE15	**0.56**	0.04	0.13	–0.01
FMPS	I never feel like I can meet my parents’ expectations.	PC22	**0.50**	–0.31	0.35	–0.08
CAPS	My teachers expect my work to be perfect.	SPP21	**0.48**	0.14	0.05	0.05
CAPS	People around me expect me to be great at everything.	SPP15	**0.48**	0.29	0.06	0.01
FMPS	I am punished for doing things less than perfect	PC3	**0.47**	–0.17	0.10	0.03
FMPS	My parents never try to understand my mistakes.	PC5	**0.40**	–0.13	0.08	–0.14
CAPS	Other people think that I have failed if I do not do my very best all the time.	SPP12	**0.38**	0.13	0.18	–0.08
CAPS	I want to be the best at everything I do.	SOP2	–0.02	**0.86**	–0.16	–0.12
CAPS	I try to be perfect in everything I do.	SOP1	–0.02	**0.76**	–0.13	0.04
CAPS	I don’t always try to be the best	SOP10	–0.22	**0.74**	–0.04	–0.19
FMPS	I set higher goals than most people.	PS12	–0.10	**0.72**	–0.02	0.15
FMPS	I don’t always try to be the best.	PS6	0.08	**0.69**	0.03	0.03
CAPS	I do not have to be the best at everything I do.	SOP22	–0.04	**0.62**	0.02	–0.38
CAPS	I always try to be as perfect as I can	SOP14	0.15	**0.62**	–0.06	0.09
CAPS	When I do something, it has to be perfect	SOP16	0.10	**0.62**	0.15	–0.03
CAPS	I get upset if there is even one mistake in my work	SOP11	0.03	**0.60**	0.19	–0.18
FMPS	I have extremely high goals.	PS19	0.12	**0.60**	–0.13	0.26
CAPS	I feel that I have to do my best all the time.	SOP4	0.18	**0.55**	–0.02	0.07
FMPS	I expect higher performance in my daily tasks than most people.	PS30	–0.05	**0.51**	0.24	0.18
CAPS	It really bothers me if I don’t do my best all the time.	SOP7	0.04	**0.51**	0.16	0.15
FMPS	Other people seem to accept lower standards than I do.	PS24	–0.21	**0.44**	0.25	0.14
CAPS	I can’t stand to be less than perfect.	SOP17	0.09	**0.43**	0.25	–0.14
FMPS	If someone does a task at school better than I am, then I feel like I failed the whole task.	CM13	–0.04	0.04	**0.76**	0.06
FMPS	I usually have doubts about the simple everyday things I do.	DA28	–0.16	–0.10	**0.73**	0.19
FMPS	It takes me a long time to do something “right.”	DA33	–0.04	–0.15	**0.69**	–0.04
FMPS	If I do not do well all the time, people will not respect me.	CM25	0.10	–0.08	**0.64**	0.06
FMPS	If I fail partly, it is as bad as being a complete failure.	CM14	–0.05	0.14	**0.62**	0.00
FMPS	Even when I do something very carefully, I often feel that it is not quite right.	DA17	0.05	0.02	**0.57**	0.13
^∗^CAPS	Even when I pass, I feel that I have failed if I didn’t get one of the highest marks in the class.	*SOP19*	–0.07	0.33	***0.53***	0.01
FMPS	If I fail at school, I am a failure as a person.	CM9	0.09	0.15	**0.53**	–0.05
FMPS	The fewer mistakes I make, the more people will like me.	CM34	0.23	–0.01	**0.51**	–0.03
FMPS	I tend to get behind in my work because I repeat things over and over.	DA32	0.03	0.00	**0.50**	0.00
FMPS	If I do not as well as other people, it means I am an inferior human being.	CM23	0.18	0.03	**0.49**	–0.15
FMPS	People will probably think less of me if I make a mistake.	CM21	0.08	0.21	**0.48**	–0.12
FMPS	I am an organized person.	Org31	–0.04	–0.03	0.03	**0.83**
FMPS	I am a neat person.	Org7	0.06	–0.11	–0.10	**0.79**
FMPS	I try to be an organized person.	Org8	0.01	0.01	–0.05	**0.79**
FMPS	Organization is very important to me.	Org2	0.02	–0.35	0.18	**0.75**
FMPS	I try to be a neat person.	Org27	0.09	–0.01	–0.05	**0.68**
FMPS	Neatness is very important to me.	Org29	0.02	0.08	0.20	**0.65**
^∗^FMPS	I am very good at focusing my efforts on attaining a goal.	*PS16*	–0.06	*0.31*	0.00	***0.52***
^∗^CAPS	I always try for the top score on a test.	*SOP6*	–0.17	*0.23*	–0.01	***0.43***
	Eigenvalues		13.54	6.00	2.49	2.23
	% of explained variance		26.04	11.53	4.78	4.28

*CMDA = Concerns Over Mistakes and Doubts About Actions, O = Organization, SOPS = Self-Oriented Perfectionism-Personal Standards, SPPEC = Socially Prescribed Perfectionism-Parental Expectations and Criticism. ^∗^Items loading onto unexpected components (SOP19, PS16, SOP6). The bold font highlights that the item has its highest loading corresponding to component 1, 2, 3, or 4.*

## Confirmatory Factor Analyses

The joint perfectionism factor model generated by the PCA from the first sample split was cross-validated on the second sample split, and additionally compared to the following competing models: (1) a simple one-factor model (Model 0), (2) the original FMPS and CAPS specified as eight (six + two) primary correlated factors (Model 1), and (3) the current joint four-factor (Model 2). As expected models 1 and 2 performed better than Model 0, and model 1 fitted better than model 2, given the more nuanced item covariance modeling (Table 3). The performance of Model 2 (four factors) was close to Model 1 (eight factors) in terms of absolute and relative fit given the substantial reduction in model complexity, which speaks for retaining Model 2 for parsimonious reasons. The relative fit indices (CFI and TLI) of Model 2 were unsatisfactorily low, whereas the more important model misspecification index (RMSEA) was within an acceptable region. Although keeping in mind that the RMSEA tends to over-perform more complex models (Fan and Sivo, 2007), as Model 2 is an example of, it does not invalidate the main objective of finding the most parsimonious and theoretically meaningful model for the final profiling of perfectionism. The factor scores of Model 2 was saved and used in the LCA analyses.

**TABLE 3:** Confirmatory Factor Analysis of the F-MPS and the CAPS.

	**χ^2^**	***df***	**MLR scaling correction**	**CFI**	**TLI**	**RMSEA**	**SRMR**
Model 0 – 1 factor (57 items)	5880	1539	1.070	0.55	0.53	0.082	0.103
Model 1 – 8 factors (6 FMPS + 2 CAPS)	3245	1511	1.060	0.81	0.81	0.053	0.074
Model 3 – 4 factors (49 items)	2704	1121	1.075	0.80	0.79	0.058	0.071

*χ^2^, square difference test; *df*, degrees of freedom; MLR scaling correction, Scaling Correction Factor for MLR; CFI, comparative fit index; TLI, tucker-lewis index; RMSEA, root mean square error of approximation; SRMR, standardized root mean square residual.*

### Latent Class Analysis – Profiles of Perfectionism

**FIGURE 1:**
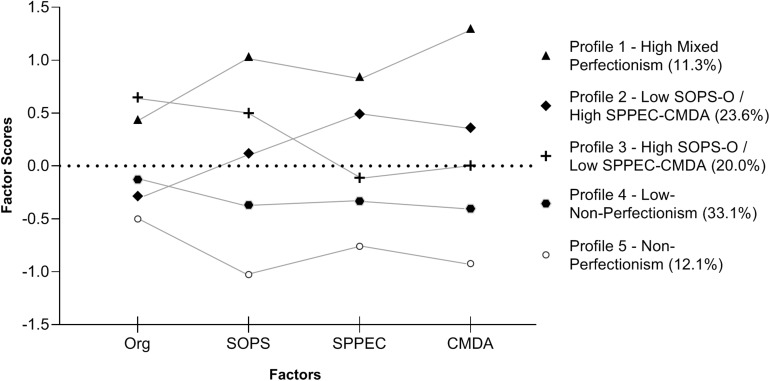
The five profiles of perfectionism and the corresponding four factors, factor mean scores. CMDA, Concerns Over Mistakes and Doubts About Actions; O, Organization; SOPS, Self-Oriented Perfectionism-Personal Standards; SPPEC, Socially Prescribed Perfectionism-Parental Expectations and Criticism.

Three of the variances were estimated as free (i.e., SPPEC, CMDA and O) in the LCA analyses as the BIC/aBIC was markedly worse if constrained as equal. The fourth variance (SOPS) was kept equal as it varied little between the classes and the change in BIC/aBIC was minor if free. The modeling started with one class and increased continually until model fit did not improve (Table 4). The improvement in fit stopped after nine classes according to BIC. Since the interpretation of an LCA analysis swiftly becomes complex if fitting too many classes, we evaluated the rate of improvement in model fit (reduction in BIC/aBIC). We preferred a solution that showed a clear deceleration in the improvement of fit (akin to the scree-plot criterion), which led us to retain five classes (Figure 1). This is also a reasonable number of classes to interpret and analyze further, as presented in Table 5.

**TABLE 4:** Fit Indices for Twelve Latent Class Models.

**Latent Classes**	**LL**	**AIC**	**BIC**	**ΔBIC**	**aBIC**	**ΔaBIC**	**Entropy**
1	–3469.54	6955.08	6992.87		6967.46		–
2	–2971.61	5975.21	6050.79	–942.08	5999.98	–967.48	0.793
3	–2799.24	5646.47	5759.85	–290.94	5683.63	–316.35	0.798
4	–2699.50	5464.00	5614.16	–145.69	5512.54	–171.09	0.803
5	–2615.04	5310.07	5499.02	–115.14	5372.00	–140.54	0.795
6	–2566.39	5228.78	5455.52	–43.50	5303.09	–68.91	0.805
7	–2531.77	5175.54	5440.08	–15.44	5262.24	–40.85	0.766
8	–2494.37	5116.74	5419.06	–21.02	5215.82	–46.42	0.789
9	–2465.72	5075.45	5415.56	–3.50	5186.91	–28.91	0.816
10	–2438.86	5037.71	5415.62	0.06	5161.57	–25.34	0.799

*LL, log likelihood ratio; AIC, Akaike’s information criterion; BIC, bayesian information criterion, ΔBIC, change in BIC; aBIC, adjusted Bayesian information criterion; ΔaBIC, change in aBIC.*

**TABLE 5:** Five profiles of perfectionism from the latent class analyses.

**Five profiles of perfectionism:**	**Description**
(1) High Mixed Perfectionism	High ratings on all four dimensions of perfectionism, i.e., exceedingly high personal standards and a need to fulfill them, with a conviction that others requires perfection, and a personal concern and doubt about own performances. In addition, organization, order and neatness are emphasized.
(2) Low SOPS-O/High SPPEC and CMDA	Being more concerned and doubtful about whether one meets the requirements of perfectionism from others, but does not set exceedingly high personal performance standards or emphasize organization, order and neatness.
(3) High SOPS-O/Low SPPEC and CMDA	Setting personal performance standards and emphasizes organization, order and neatness, but no experience that significant others have high expectations of one’s performances, and is not highly concerned and doubting own performances.
(4) Low/Non- Perfectionism	This profile indicates low personal standards, no experience of high expectations from others, and negligible concerns and doubts about personal performances.
(5) Non-Perfectionism	Similar to profile 4, but with even lower scores on all four dimensions of perfectionism.

*CMDA, concerns over mistakes and doubts about actions; O, organization; SOPS, self-oriented perfectionism-personal standards; SPPEC, socially prescribed perfectionism-parental expectations and criticism.*

### The Proportion of Girls and Boys From Specialized- and Ordinary Schools Within Each of the Profiles of Perfectionism

More girls (15.8%) compared to boys (6.2%) were observed within profile 1 (high mixed perfectionism). Furthermore, a higher relative proportion of ordinary school girls (39.3%) compared to elite school girls (25.6%), and ordinary school boys (36.4%) compared to elite school boys (19%), were observed within profile 1 and profile 2 (low self-oriented perfectionism with high perfectionistic concerns), which were the two profiles hypothesized to be associated with the most debilitating health outcomes. A higher proportion of the elite school students had a profile of higher personal standards and lower external fears, concerns and doubts related to their performance (Profile 3) compared to students in ordinary schools (Table 6).

**TABLE 6:** Proportions of perfectionism profiles within school setting and gender.

	**Profiles**	**(1)**		**(2)**		**(3)**		**(4)**		**(5)**		**Chi-square**	
		**High mixed**		**Low SOPS-O/high**		**High SOPS/low**		**Low-non-**		**Non-**		**tests**	
		**perfectionism**		**SPPEC-CMDA**		**SPPEC-CMDA**		**perfectionism**		**perfectionism**			
		***%***	***n***	***%***	***n***	***%***	***n***	***%***	***n***	***%***	***n***	**χ^2^**	***p***
GIRLS *n* = 443	Specialized¤ schools *n* = 82	*12.2*^a^	10	*13.4*^a^	11	*31.7*^a^	26	*30.5*^a^	25	*12.2*^a^	10	*10.36*	*0*.*035*
	Ordinary schools *n* = 361	*16.6*^a^	60	*22.7*^a^	82	*17.5*^b^	63	*30.5*^a^	110	*12.7*^a^	46		
BOYS *n* = 389	Specialized¤ schools *n* = 84	*6.0*^a^	5	*13.1*^a^	11	*34.5*^a^	29	*32.1*^a^	27	*14.3*^a^	12	*20.12*	*0*.*000*
	Ordinary schools *n* = 305	*6.2*^a^	19	*30.2*^b^	92	*15.7*^b^	48	*37.0*^a^	113	*10.8*^a^	33		
TOTAL *n* = 832	Girls *n* = 443	*15.8*^a^	70	*21.0*^a^	93	*20.1*^a^	89	*30.5*^a^	135	*12.6*^a^	56	*21.76*	*0*.*000*
	Boys *n* = 389	*6.2*^b^	24	*26.5*^a^	103	*19.8*^a^	77	*36.0*^a^	140	*11.6*^a^	45		

*^*a*, *b*^different lettered subscripts indicate significant difference between the school groups at *p* < *0.05* for the proportion within the perfectionism profiles. CMDA, concerns over mistakes and doubts about actions; O, organization; SOPS, self-oriented perfectionism-personal standards; SPPEC, socially prescribed perfectionism-parental expectations and criticism; ¤ Specialized schools, students attending specialized schools for talented athletes and performing artists.*

### Comparisons of the Five Perfectionism Profiles With Regard to Psychological Health

A MANOVA with five dependent criterion variables (anxiety, depression, EDE-Q weight and shape concern (WCSC), resilience and self-worth) indicated an overall significant difference between the five perfectionism profiles (*F*_20,2654_ = 16.32, *p* < *0.0001*; Wilks’ 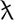 = 0.68; partial η^2^ = 0.09), which was followed up with a separate ANOVA for each outcome variable (Table 7). A MANOVA of the four subdimensions of perfectionism as dependent variables (SOPS, SPPEC, CMDA and O) also indicated an overall significant difference between the five profiles (*F*_16,2454_ = 183.96, *p* < 0.0001*; Wilks’* 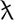 = *0.09;* partial η2 = 0.45). The follow-up ANOVA confirmed differences between the profiles for all criterion variables: Anxiety, *F*_4,817_ = 49.78, partial η2 = 0.20, Depression, *F*_4,819_ = 55.99, partial η2 = 0.22, EDE-Q WCSC, *F*_4,824_ = 23.82, partial η2 = 0.10, Resilience, *F*_4,818_ = 32.80, partial η2 = 0.14, Self-Worth, *F*_4,821_ = 36.47, partial η2 = 0.15, *Ps* < *0.0001*. Scheffe’s multiple comparisons are presented in Table 7.

**TABLE 7:** The five perfectionism profiles and mean_95%CI_ on the criterion related variables of the revised anxiety depression scale (RCADS), the eating disorder examination questionnaire (EDE-Q) weight-concern and shape-concern, self-worth (SPPA-R), and resilience scale for adolescents (READ).

	**(1)**		**(2)**		**(3)**		**(4)**		**(5)**		**Multiple**
	**High mixed**		**Low SOPS-O/high**		**High SOPS-O/Low**		**Low-non-**		**Non-**		**comparison**
	**perfectionism**		**SPPEC-CMDA**		**SPPEC-CMDA**		**perfectionism**		**perfectionism**		**between**
	**(*n* = 94)**		**(*n* = 196)**		**(*n* = 166)**		**(*n* = 275)**		**(*n*=101)**		**each profile**
	***M*_95% *CI*_**	**Rank**	***M*_95% *CI*_**	**Rank**	***M*_95% *CI*_**	**Rank**	***M*_95% *CI*_**	**Rank**	***M*_95% *CI*_**	**Rank**	***p* < 0.05**
Criterion variables											
Anxiety (crude)	17.09_15.85, 18.33_	1	11.97_11.10, 12.83_	2	9.81_8.88, 10.74_	3	8.21_7.48, 8.93_	4	6.60_5.40, 7.80_	5	1 > 2–5, 2 > 3–5, 3 > 5
Anxiety (adj.)	14.77_13.05, 16.50_		12.00_10.71, 13.28_		9.22_8.28, 10.16_		8.03_7.15, 8.91_		6.21_4.80, 7.62_		
Depression (crude)	12.05_11.14, 12.96_	1	8.76_8.13, 9.40_	2	5.88_5.20, 6.57_	3	5.42_4.89, 5.95_	4	4.50_3.63, 5.38_	5	1 > 2–5, 2 > 3–5
Depression (adj.)	9.71_8.43, 10.99_		8.89_7.94, 9.84_		5.69_4.99, 6.38_		5.22_4.57, 5.87_		4.69_3.67, 5.71_		
WCSC (crude)	2.51_2.21, 2.82_	1	1.98_1.77, 2.19_	2	1.31_1.08, 1.54_	3	1.15_0.98, 1.33_	4	0.94_0.65, 1.24_	5	1 > 3–5, 2 > 3–5
WCSC (adj.)	1.77_1.37, 2.17_		1.86_1.56, 2.16_		1.12_0.90, 1.34_		1.04_0.83, 1.24_		0.92_0.60, 1.24_		1 > 2–5, 2 > 3–5
Resilience (crude)	3.73_3.63, 3.84_	2	3.66_3.59, 3.73_	1	4.23_4.15, 4.31_	5	4.00_3.94, 4.06_	3	4.03_3.93, 4.13_	4	1 < 3–5, 2 < 3–5, 3 > 4–5
Resilience (adj.)	3.90_3.75, 4.05_		3.65_3.54, 3.77_		4.25_4.17, 4.33_		4.00_3.92, 4.08_		4.04_3.92, 4.16_		
Self-worth (crude)	2.66_2.53, 2.79_	1	2.94_2.85, 3.03_	2	3.33_3.24, 3.43_	3	3.37_3.29, 3.44_	4	3.46_3.33, 3.58_	5	1 < 2–5, 2 < 3–5
Self-worth (adj.)	3.02_2.84, 3.20_		2.95_2.82, 3.08_		3.40_3.30, 3.50_		3.40_3.31, 3.49_		3.49_3.35, 3.64_		
Overall rank		1		2		3		4		5	

*Cut-off scores for; RCADS anxiety: girls = 26, boys = 22, RCADS depression: girls = 17, boys = 16 (Ebesutani et al., 2012). Cut-off scores for; EDE-Q severe clinical ≥4.0, Age group 16–19: ≥2.7 (Rø et al., 2015) (No agreed cut-point exist for the age group in our study). Adj, adjusted for gender and school setting; CMDA, concerns over mistakes and doubts about actions; O, organization; SOPS, self-oriented perfectionism-personal standards; SPPEC, socially prescribed perfectionism-parental expectations and criticism; WCSC, EDE-Q weight concern and shape concern.*

Profile 1 (high mixed perfectionism), and 2 (low SOPS-O/High SPPEC-CMDA) (Table 5 and Figure 1) were associated with the highest levels on anxiety, depression and WCSC, and the lowest ratings for resilience and global self-worth (Table 7). No significant differences were found in depression, WCSC, and self-worth between the two non-perfectionism groups (profiles 4–5) and profile 3. The anxiety score was higher for profile 3 than the non-perfectionism groups, and the adolescents within profile 3 had higher resilience ratings than all other perfectionism profiles.

The interaction effect between gender, school group and perfectionism profile was not statistically significant for any of the criterion variables. Adjusting for gender and school group changed the scores for the dependent criterion variables for profile 1 only, whereas the anxiety, depression and WCSC decreased and resilience and self-worth increased. Additionally, adjusting for gender and school group resulted in lower WCSC scores within profile 1 compared to profile 2.

## Discussion

### Profiles of Perfectionism Derived From Factor Scores of the FMPS and CAPS

The separate factor structure of the FMPS and CAPS supported previous findings (e.g., Sironic and Reeve, 2015). When the items of the two questionnaires were combined, a four-dimensional model was the most parsimonious and theoretically meaningful to use for the final profiling of perfectionism. The subsequent LCA yielded five distinct profiles of perfectionism. Compared with a solution with four and six profiles, this five-profile solution fitted the data better (Table 4), and it was used in the further analyses as the most reasonable model to interpret. Moreover, this solution aligns with a consistent pattern of perfectionism among adolescents reported in previous studies (Dixon et al., 2004; Boone et al., 2010; Hill, 2013; Sironic and Reeve, 2015). Notably, the present study identified one ‘high mixed’ perfectionism profile (Profile 1) with combined high levels of all four factors except for “organization.” Profile 2 may reflect a tendency of perceiving standards originated from other people (Sironic and Reeve, 2015), and that a failure to meet such standards and expectations may elicit disapproval, criticisms or even rejection (Frost et al., 1990). Some (Stoeber and Otto, 2006; Stoeber, 2018a) argue that the external facets of ‘parental expectation’ and ‘parental criticism’ from FMPS (Frost et al., 1990) should rather be considered as antecedents of perfectionism. However, young adolescents like in the present study are in a developmental stage where they are perceptive and thus, vulnerable to perceived external standards and pressure to conform with them (Hall and Hill, 2012; Bergeron et al., 2015; Flett et al., 2016; Curran, 2018). Hence, such external factors may affect how an adolescent think and behave at school or in competitive contexts.

The third profile mirrors a previously proposed “adaptive” profile of perfectionism (Sironic and Reeve, 2015) or the “pure personal standards perfectionism” in the 2 × 2 model of perfectionism (Gaudreau and Thompson, 2010; Hill, 2013). The present findings suggest that a subgroup of young adolescents do not display a perfectionistic trait *per se* (Hill, 2016), but rather set sound personal standards with barely any perfectionistic concerns. In a similar vein and consistent with previous studies (Sironic and Reeve, 2015) another group of adolescents was identified by profiles 4 and 5, in which aspects of perfectionism were of negligible or no relevance. In total, our findings support the notion of individual differences in how perfectionism may operate (Gaudreau and Thompson, 2010; Sironic and Reeve, 2015) as well as the interaction of individual and interpersonal components that may affect adolescents’ health and well-being (Hall et al., 2012; Hewitt et al., 2017).

### The Proportion of Girls and Boys From Specialized Schools and Ordinary Schools Within Each of the Profiles of Perfectionism

The relative proportion of adolescent who are really plagued with perfectionism (profile 1 and 2) were lower in elite sports- and performing arts schools (22%) than ordinary schools (38%). This might seem contra-intuitive given the considerable amount of time sports- and performing arts school students spend in a highly competitive context. However, contextual and selection issues may account for the fact that more young girls and boys from elite schools do seem to set high personal standards, yet they do not experience highly doubts about their performances or external pressure or expectations. For instance, many students in ordinary schools may experience a distance between their capacities and external standards or demands. Furthermore, those who attend elite schools have actively sought such schools and passed the admittance criteria that they experience as reasonable and achievable. Moreover, at high performance levels, an internalization of high standards and goals are necessary and may serve as driving factors to reach further development and achievements (Hall and Hill, 2012; Hill et al., 2015). Yet, attention toward external performance pressures, and on holding realistic personal standards and goals, should also be a focus in elite schools (Bergeron et al., 2015).

### Identified Perfectionism Profiles and Mental Health and Psychological Functioning

The present study showed that profile 1 and 2 were related to significantly higher levels of anxiety, depression and excessive weight and shape concerns as well as lower levels of resilience and self-worth (Table 7). These findings add support to previous studies (Sironic and Reeve, 2015) showing that adolescents who may fit into profile 1 and 2 may be more vulnerable to mental health problems and that higher combined perfectionism levels (Profile 1) may endanger mental health (Boone et al., 2010; Gustafsson et al., 2016). Of note, a rather low psychological burden seems to be present in the large group of adolescents who display moderate self-oriented strivings in addition to experiencing low external pressure (Profile 3) (Dixon et al., 2004). This finding supports the understanding that mental health problems related to perfectionism relate to the self-critique and the overly evaluative processes and not to holding personal standards for performance or actions *per se* (Hill, 2013, 2016).

Our findings adds further support to study the interaction of facets of perfectionism, because the within-person levels of perfectionism, which differs between the five profiles, are differently related to the criterion variables. Moreover, even though the two “non-perfectionism” profiles may have limited practical relevance, the findings of the overall outcomes of all five perfectionism profiles (Table 7) suggest that there is a pattern of the profiles, from profile 1 to profile 5, which is successively linked to worse or better scores on the mental health variables. Hence, these findings indicate that the lower the overall perfectionism scores are, the better the adolescents score on the mental health outcome measures (except from resilience which were highest within profile 3).

Moreover, the interaction effect between perfectionism profiles, gender and school group (i.e., “specialized school” and “ordinary school”) was not statistically significant for any of the dependent criterion variables (Table 7). Thus, at this age, specialized school settings may not be the prime target for overall actions against sources and consequences of perfectionism. However, the potential downsides of perfectionism are detrimental, and adolescents in a developmental age in both specialized and ordinary school settings, as in the present study, are vulnerable (Bergeron et al., 2015; Flett et al., 2016; Curran, 2018).

### Implications, Strengths, Limitations, and Future Research

Our results indicate a prevalence of perfectionistic tendencies that is on par with international trends (Sironic and Reeve, 2015; Curran and Hill, 2017), and highlight a need of attention toward lowering external performance pressure and personal quality standards at variance with realistic goals in order to reduce the risk of adjustment difficulties and mental health problems (Bergeron et al., 2015).

Several strengths of this study comprise the use of a sample which is large, almost equally gender distributed across very young adolescents within both ordinary and elite performance contexts. Moreover, the total number of “elite” specialized lower secondary schools in Norway were included in our study. This strength also represents a limitation as it was not possible to increase this subsample to match the ordinary school sample. As a result, the absolute number of subjects in some of the profiles may be considered as suboptimal for generalization purposes and for the purpose of robust comparisons of relative proportions between the school groups and genders. This is, however, the first study that compares perfectionism among younger adolescents who attend both specialized sports-/performing arts and regular schools.

Measures of resilience and self-worth included as criterion variables is a strength, and extend previous findings regarding associations between combined facets of perfectionism levels and poor health indicators. On the other hand, subgroups across studies will probably diverge (Stoeber, 2018b), preventing a direct comparison of latent classes (profiles). However, comparable perfectionism profiles like in the present study have previously been, and may in forthcoming studies be, identified by others when utilizing a person-centered approach (Sironic and Reeve, 2015). Recognizing the perils of cross-sectional data, more research is needed to explore stability or change in perfectionism profiles over time. Such issues will be examined in forthcoming longitudinal studies of the present material, and with the potential of person-oriented interventions to prevent the potential downsides of perfectionism among young adolescents in a vulnerable developmental stage of life.

## Data Availability

All datasets generated for this study are included in the manuscript and/or the Supplementary Files.

## Ethics Statement

The protocol of the study was approved by the Regional Committee for Medical and Health Science Research Ethics (REC) in Southern Norway (project nr.2015/1358). The study was carried out in accordance with the recommendations of the ethical guidelines, health research legislations and regulations (The Health Research Act, 2008; Regulations on the organization of medical and health research, 2009; the Personal Data Act, 2000; Act on ethics and integrity in research, 2017). All participants gave written informed consent in accordance with the Declaration of Helsinki.

## Author Contributions

AS and JS-B conceived the study. All authors contributed to the development of the design and manuscript revision, and have read and approved the submitted version. AS collected and organized the data and performed the statistical analysis with major contributions from OF. AS wrote the first draft of the manuscript.

## Conflict of Interest Statement

The authors declare that the research was conducted in the absence of any commercial or financial relationships that could be construed as a potential conflict of interest.
