# Supplementary material for: Profiles of Perfectionism Among Adolescents Attending Specialized Elite- and Ordinary Lower Secondary Schools: A Norwegian Cross-Sectional Comparative Study
Source: Front Psychol. 2019 Sep 6;10:2039. doi: 10.3389/fpsyg.2019.02039 (PMC6743349; doi:10.3389/fpsyg.2019.02039)
Supplement: Supplementary file 1 [file Data_Sheet_1.docx]

Supplementary Material

Two tables: Principal Component Analysis of 1) the FMPS and 2) the CAPS.

# Principal Component Analysis of the Frost Multidimensional Perfectionism Scale (FMPS)

**Table X1.** Principal Component Analysis of the FMPS. Four components extracted based on the evaluation of the parallel analysis and removal of three problematic items found in step 2 of the PCA. Final solution of the FMPS PCA analysis.

| Item |  | **1**  **CMDA** | **2**  **PEPC** | **3**  **O** | **4**  **PS** |
| --- | --- | --- | --- | --- | --- |
| DA28 | I usually have doubts about the simple everyday things I do. | **0.75** | -0.23 | 0.21 | -0.08 |
| CM13 | If someone does a task at school better than I am, then I feel like I failed the whole task. | **0.69** | 0.00 | 0.06 | 0.09 |
| DA33 | It takes me a long time to do something "right." | **0.67** | -0.09 | -0.01 | -0.11 |
| CM25 | If I do not do well all the time, people will not respect me. | **0.62** | 0.06 | 0.04 | 0.04 |
| CM14 | If I fail partly, it is as bad as being a complete failure. | **0.60** | -0.03 | -0.01 | 0.16 |
| CM21 | People will probably think less of me if I make a mistake. | **0.59** | 0.00 | -0.15 | 0.23 |
| DA17 | Even when I do something very carefully, I often feel that it is not quite right. | **0.58** | -0.01 | 0.04 | 0.15 |
| CM34 | The fewer mistakes I make, the more people will like me. | **0.57** | 0.14 | -0.06 | 0.04 |
| CM9 | If I fail at school, I am a failure as a person. | **0.53** | 0.14 | -0.06 | 0.13 |
| CM23 | If I do not as well as other people, it means I am an inferior human being. | **0.53** | 0.14 | -0.14 | 0.07 |
| DA32 | I tend to get behind in my work because I repeat things over and over. | **0.51** | 0.08 | 0.06 | -0.06 |
| PE20 | My parents have expected excellence from me. | -0.15 | **0.82** | 0.10 | 0.13 |
| PE11 | My parents wants me to be the best at everything. | -0.15 | **0.81** | -0.05 | 0.19 |
| PE1 | My parents set very high standards for me. | -0.10 | **0.79** | 0.09 | 0.16 |
| PE26 | My parents have always had higher expectations for my future than I have. | 0.03 | **0.72** | -0.09 | -0.13 |
| PE15 | Only outstanding performance is good enough in my family. | 0.06 | **0.69** | 0.03 | 0.07 |
| PC35 | I never feel like I can meet my parents' standards. | 0.25 | **0.59** | -0.02 | -0.32 |
| PC22 | I never feel like I can meet my parents' expectations. | 0.34 | **0.50** | 0.01 | -0.32 |
| PC3 | I am punished for doing things less than perfect | 0.04 | **0.43** | -0.05 | 0.01 |
| PC5 | My parents never try to understand my mistakes. | 0.09 | **0.42** | 0.00 | -0.23 |
| Org31 | I am an organized person. | -0.01 | -0.03 | **0.83** | 0.06 |
| Org8 | I try to be an organized person. | -0.05 | 0.00 | **0.79** | 0.03 |
| Org2 | Organization is very important to me. | 0.12 | -0.04 | **0.78** | -0.27 |
| Org7 | I am a neat person. | -0.14 | 0.02 | **0.74** | 0.06 |
| Org27 | I try to be a neat person. | -0.01 | 0.05 | **0.71** | 0.02 |
| Org29 | Neatness is very important to me. | 0.20 | 0.01 | **0.65** | 0.11 |
| PS12 | I set higher goals than most people. | 0.00 | -0.07 | -0.11 | **0.95** |
| PS19 | I have extremely high goals. | -0.08 | 0.12 | 0.06 | **0.81** |
| PS24 | Other people seem to accept lower standards than I do. | 0.18 | -0.14 | -0.10 | **0.70** |
| PS30 | I expect higher performance in my daily tasks than most people. | 0.28 | -0.01 | 0.01 | **0.67** |
| PS6 | I don’t always try to be the best. | 0.11 | 0.19 | 0.10 | **0.52** |
| PS16 | I am very good at focusing my efforts on attaining a goal. | -0.04 | -0.01 | 0.32 | **0.50** |
|  | Eigenvalues | 7.78 | 4.92 | 1.92 | 1.72 |
|  | % of variance | 24.32 | 15.39 | 6.00 | 5.37 |

***Note.*** CM = Concern over Mistakes. DA = Doubts About Actions. O = Organization. PE = Parental Expectations. PC = Parental Criticism. PS = Personal Standards.

Three items were removed before the final PCA was performed due to loadings onto unexpected components. PS 4 ("If I do not set the highest standards for myself, I am likely to end up a second-rate person") had a low loading on the expected component (i.e.,0.28) and loaded onto the CMDA component. The CM items 10 ("I should be upset if I make a mistake.") and 18 ("I hate being less than the best at things.") loaded onto the PS component and had a low loading on the expected components (i.e., 0.19 and -0.05, respectively).

# Principal Component Analysis of the Child Adolescent Perfectionism Scale (CAPS).

**Table X2.** CAPS component pattern matrix. Two components extracted based on the evaluation of the parallel analysis and removal of two problematic items found in step 2 of the PCA. Final solution of the CAPS PCA analysis.

| Item |  | **1**  **SPP** | **2**  **SOP** |
| --- | --- | --- | --- |
| SPP5 | There are people in my life who expect me to be perfect. | **0.88** | -0.13 |
| SPP13 | Other people always expect me to be perfect. | **0.84** | -0.06 |
| SPP9 | People expect more from me than I am able to give. | **0.84** | -0.18 |
| SPP8 | My family expects me to be perfect. | **0.77** | -0.06 |
| SPP3 | I feel that people ask too much of me. | **0.66** | 0.07 |
| SPP15 | People around me expect me to be great at everything. | **0.62** | 0.18 |
| SPP12 | Other people think that I have failed if I do not do my very best all the time. | **0.61** | 0.03 |
| SPP21 | My teachers expect my work to be perfect. | **0.60** | 0.04 |
| SOP2 | I want to be the best at everything I do. | -0.11 | **0.80** |
| SOP1 | I try to be perfect in everything I do. | -0.07 | **0.75** |
| SOP7 | It really bothers me if I don’t do my best all the time. | 0.08 | **0.69** |
| SOP10 | I don’t always try to be the best. | -0.27 | **0.68** |
| SOP14 | I always try to be as perfect as I can | 0.11 | **0.66** |
| SOP16 | When I do something, it has to be perfect. | 0.25 | **0.60** |
| SOP6 | I always try for the top score on a test. | -0.31 | **0.56** |
| SOP4 | I feel that I have to do my best all the time. | 0.22 | **0.55** |
| SOP11 | I get upset if there is even one mistake in my work | 0.17 | **0.53** |
| SOP19 | Even when I pass, I feel that I have failed if I didn’t get one of the highest marks in the class. | 0.20 | **0.49** |
| SOP22 | I do not have to be the best at everything I do. | 0.01 | **0.46** |
| SOP17 | I can’t stand to be less than perfect. | 0.28 | **0.42** |
|  | Eigenvalues | 6.96 | 2.47 |
|  | % of variance | 34.77 | 12.32 |

***Note.*** SOP = Self-Oriented Perfectionism. SPP = Socially Prescribed Perfectionism.

Two items were removed before the final PCA analysis of the CAPS. SPP 18 ("I am always expected to do better than others") due to substantial cross loading onto the second component (i.e., 0.43 versus 0.37 on its expected component), and SPP 20 ("My parents don’t always expect me to be perfect in everything I do") had a low loading on its expected component (i.e., -0.14).
